# Spinal neurenteric cyst in a dog

**DOI:** 10.1186/s13620-015-0037-2

**Published:** 2015-05-22

**Authors:** François-Xavier Ferrand, Paul Pillard, Claude Carozzo, Thierry Marchal, Marie-José Seurin, Catherine Escriou

**Affiliations:** Small Animal Surgery Department, Vet Agro Sup, Campus Vétérinaire de Lyon. 1 Avenue Bourgelat, 69280 Marcy l’Etoile, France; Histopathology Department, Vet Agro Sup, Campus Vétérinaire de Lyon. 1 Avenue Bourgelat, 69280 Marcy l’Etoile, France; Centre d’imagerie par resonance magnétique, CIRMA, 1 Avenue Bourgelat, 69280 Marcy l’Etoile, France; Small Animal Internal Medicine Department, Vet Agro Sup, Campus Vétérinaire de Lyon. 1 Avenue Bourgelat, 69280 Marcy l’Etoile, France

**Keywords:** Cyst, Spine, Dog, MRI

## Abstract

A 2-year-old female crossbreed dog was presented with progressive ataxia and paraparesis. A T3-L3 spinal lesion was determined by neurological examination. Magnetic resonance imaging (MRI) revealed an ovoid-shaped, well-circumscribed mass affecting the spinal cord at the level of the T9 vertebra. A left hemilaminectomy and a durotomy at the level of T9 allowed discovery of an ovoid deformation of the meninges with a cystic appearance.

En bloc removal was performed and appeared to be complete. Pathological analysis showed a voluminous cystic lesion lined by a heterogeneous epithelium. Three types of epithelium were present: a pseudostratified columnar epithelium, a stratified squamous epithelium and a transitional epithelium. Mucus production, the morphology of some cells with microvilli at the apical pole and immunohistochemical assays were highly in favor of an endodermal origin of the cyst. The age of the dog, anamnesis, MRI study and histological findings were consistent with an intradural neurenteric cyst as described in humans. Total surgical removal led to a progressive clinical improvement with no recurrence at 18 months. We report an unusual intradural extramedullary cyst, called a neurenteric cyst, in a 2-year-old female crossbreed dog. This type of cyst is well-known in humans but has never been described in dogs. We propose that neurenteric cysts should be included in the differential diagnoses for tumor-like or cystic intradural lesions in the young dog. Prognosis for this type of cyst seems to be good, as total surgical removal led to a progressive clinical improvement with no recurrence at 18 months.

## Background

Thoraco-lumbar intradural extra-axial myelopathies are uncommon in dogs, especially in young dogs. Pseudocysts and embryonic tumors represent the most common lesions, often causing progressive onset of clinical signs such as paraspinal hyperesthesia, ataxia, paresis, and plegia [1, 2]. True cystic structures are rare. We describe an unusual intradural extramedullary cyst in a 2-year-old female crossbreed dog. Due to its similarity to human findings, a neurenteric cyst is suspected.

## Case presentation

**Fig. 1:**
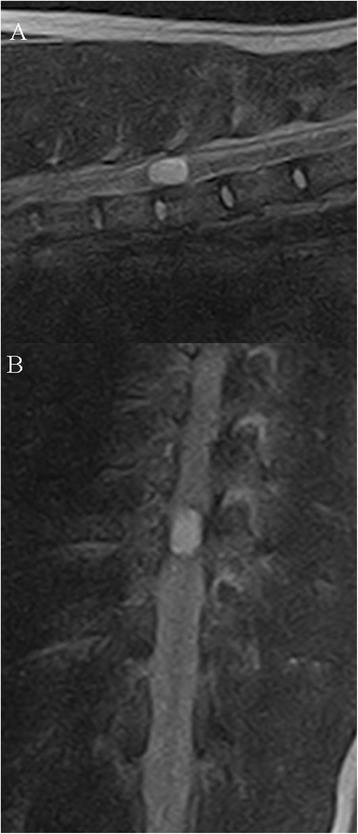
Sagittal (**a**) and dorsal (**b**) T2-weighted MRI slices of the intradural lesion

**Fig. 2:**
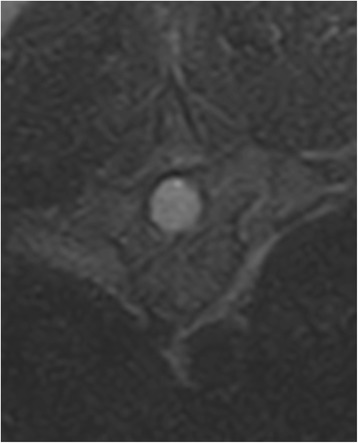
Transverse T2-weighted slice of the lesion involving the whole diameter of the spinal cord. No relation with the nerve roots was seen

A 2-year-old female mixed-breed dog (weight 25 kg) was admitted for slowly progressive ataxia and paraparesis over the course of one month. A rapid neurological deterioration occurred the week before presentation, characterized by fecal and urinary incontinence. The dog received prednisolone therapy, leading only to a minor improvement of neurological signs. On presentation, the dog showed no signs of pain. The dog was ambulatory with severe hind limb ataxia and paresis. All postural reactions were absent in the hind limbs. Spinal reflexes were increased in the hind limbs. The cutaneous trunci reflex was absent bilaterally, distal to T13. Neuroanatomical diagnosis was a T3-L3 spinal lesion consistent with upper motor neuron signs. Complete blood count and serum biochemistry analysis were unremarkable. Magnetic resonance imaging (MRI) using an open permanent low-field magnet (E-SCAN XQ device (Esaote®), B_0_ = 0,18 T) revealed an ovoid-shaped, well-circumscribed mass affecting the spinal cord at the level of the T9 vertebra. The lesion was iso-intense to slightly hypo-intense on T1-weighted images, strongly hyper-intense on T2-weighted images (Fig. 1 a and b), and non-contrast enhancing. The adjacent segments of the spinal cord were swollen. The mass seemed intradural on the first transverse section, but its large size (7.3 mm diameter for 11.6 mm long), filling the whole spinal cord diameter, did not allow a clear discrimination between an intradural or intramedullary lesion. The mass did not show any relation with the adjacent spinal nerve roots on the transverse slices (Fig. 2). Neither bony nor surrounding soft tissue-associated abnormalities could be seen. Given the age of the dog, the location, the shape and the MRI features of the lesion, we hypothesized that this was a neoplastic process such as an extrarenal nephroblastoma or a cystic structure such as an epidermoid cyst. The dog underwent surgery under general anesthesia. A standard approach to the thoracic vertebrae was made through a dorsal incision. A left hemilaminectomy from the eighth to the tenth thoracic vertebrae was performed.

**Fig. 3:**
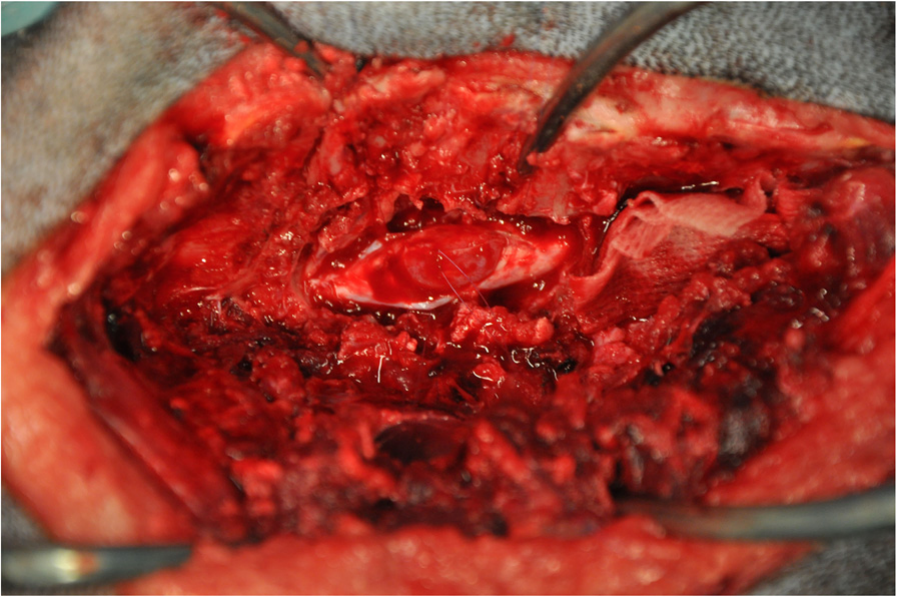
Intra-operative view of the cyst after durotomy

**Fig. 4:**
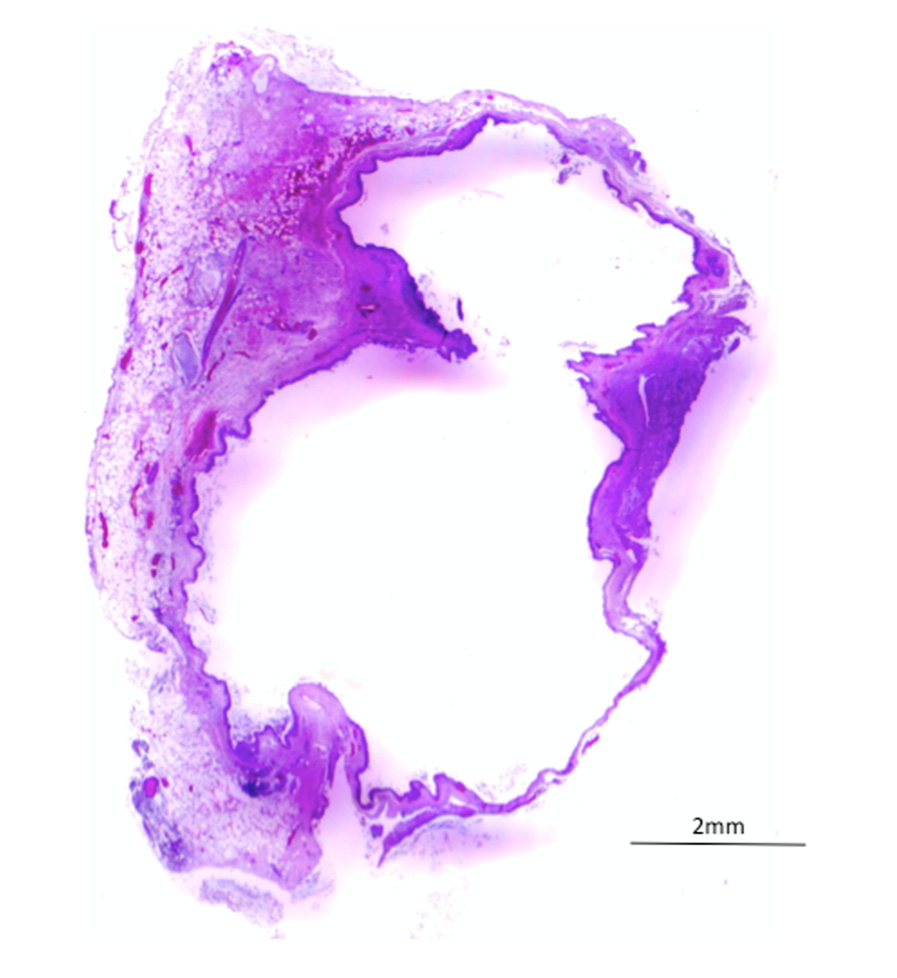
Photomicrograph of the neurenteric cyst

**Fig. 5:**
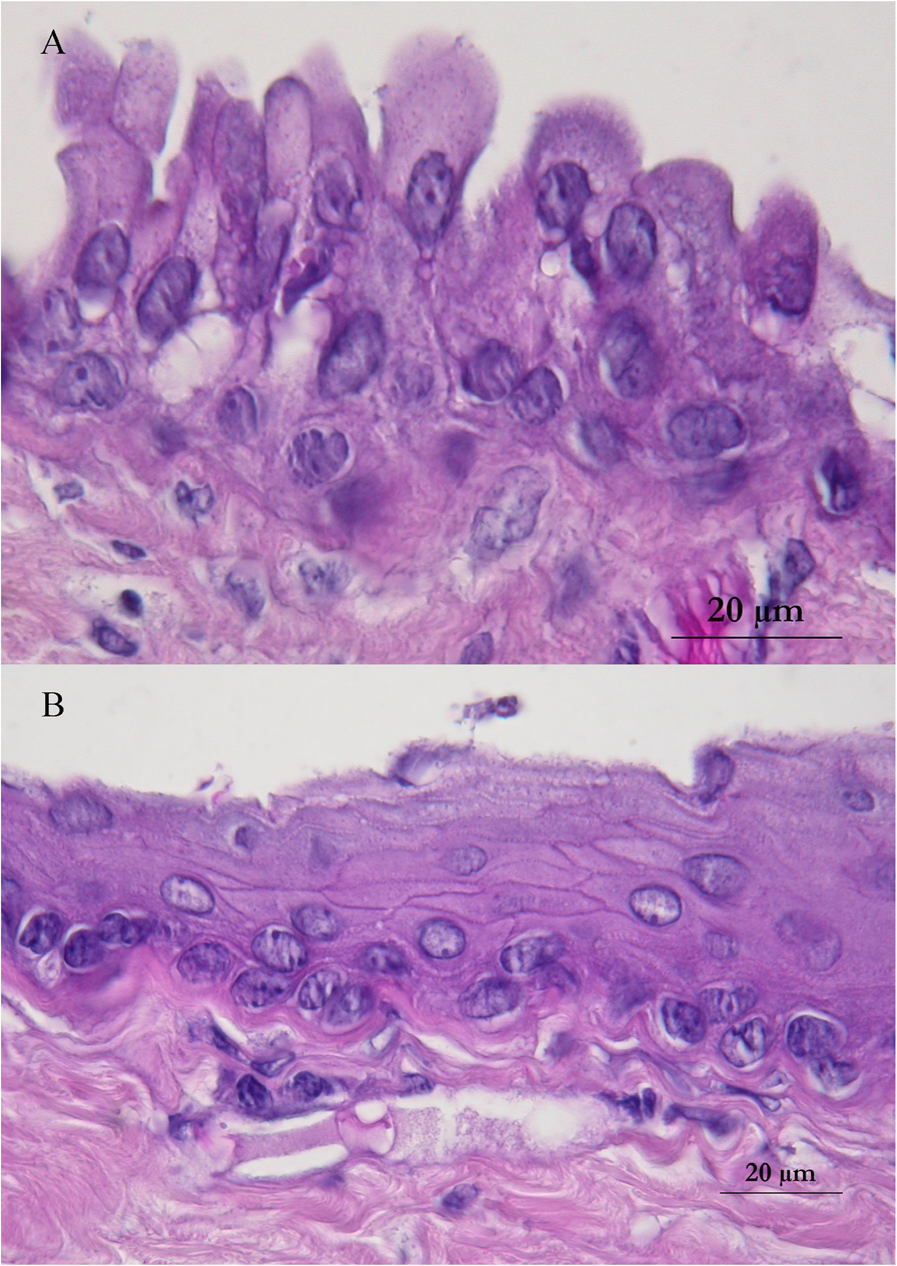
The cyst was lined by multiple epithelial structures: pseudostratified columnar epithelium with cylindrical cells with basal nuclei and round-shaped apical poles suggesting an enteral or respiratory epithelium (**a**). Stratified squamous epithelium suggesting a Malpighian epithelium but without keratin production (**b**)

**Fig. 6:**
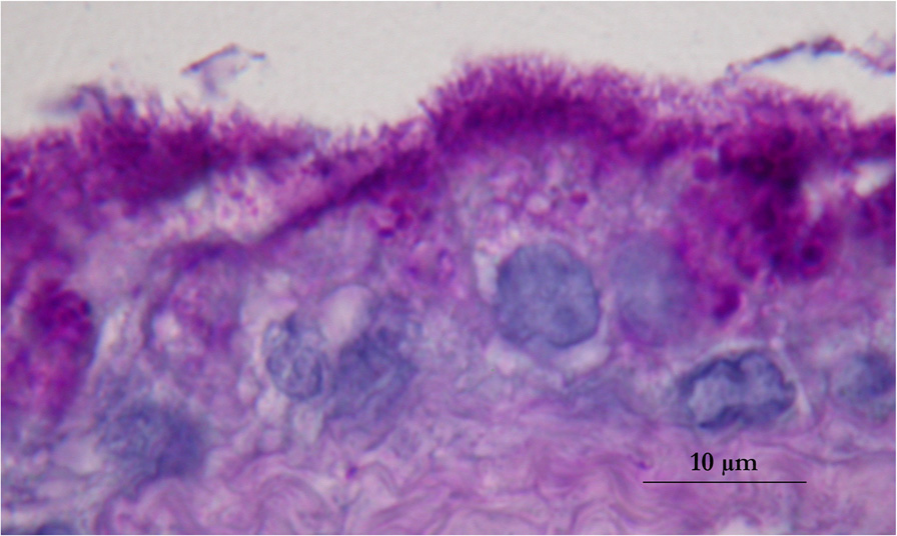
Photomicrograph showing cells with basal nucleus and filiform apical cytoplasmic extensions, which look like microvilli. Periodic acid-Schiff staining is positive which highlights the production of mucus by these cells

**Fig. 7:**
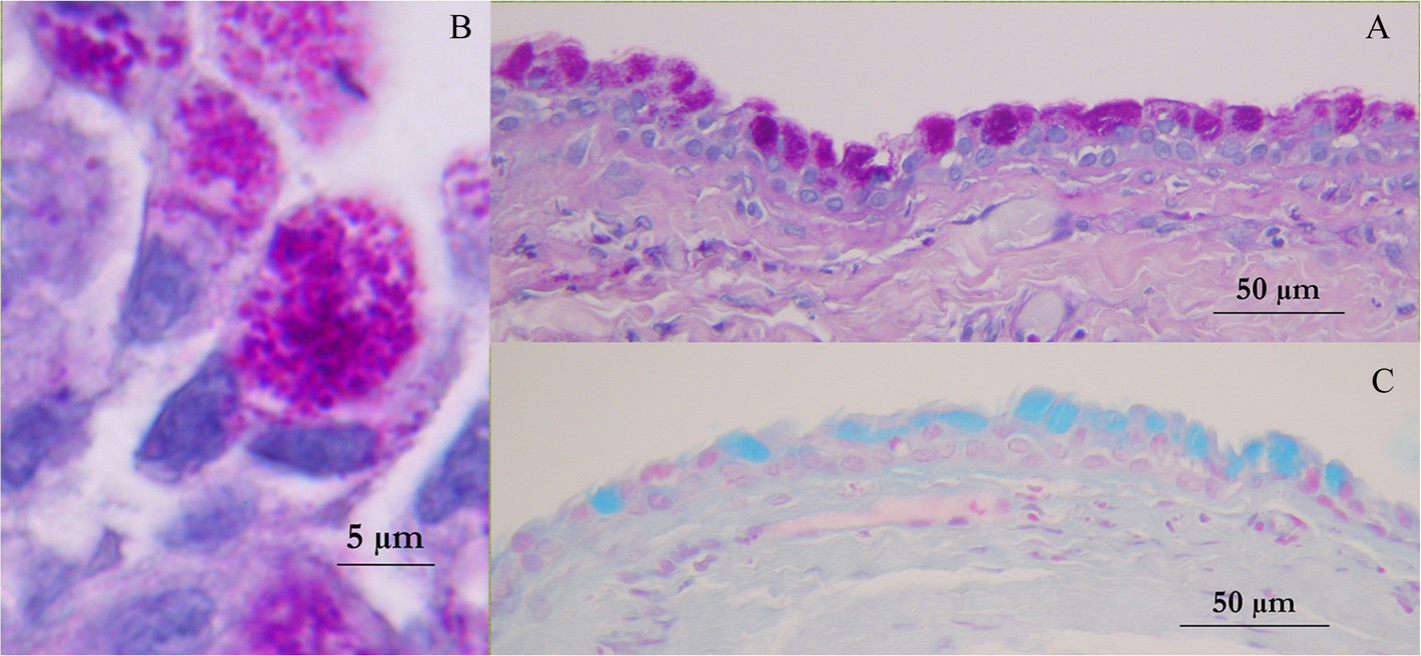
Photomicrographs showing the secretory function of the epithelium. Periodic acid-Schiff staining is positive (**a**), and highlights acidophil granules in the cytoplasm of caliciform cells with a basal nucleus and convex borders (**b**). Alcian blue staining is also positive, highlighting production of acid mucin (**c**)

**Fig. 8:**
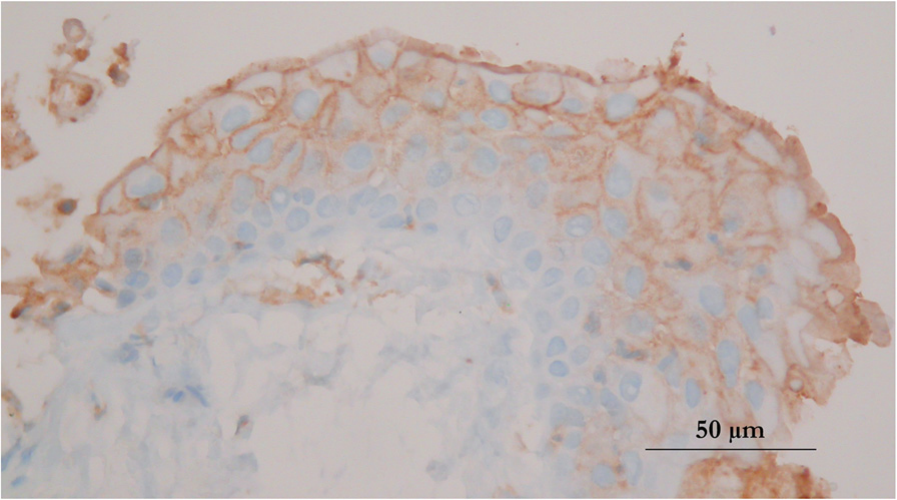
Photomicrograph showing positive staining for carcinoembryonic antigen

An ovoid deformation of the meninges with a cystic appearance could be seen at the level of T8-T9, involving the whole left side of the spine. At the dura mater opening, an intradural extramedullary soft mass could be observed (Fig. 3). A translucent, gelatinous liquid was obtained from the mass by fine needle aspiration, which looked like mucus. Dissection of the mass was then performed using microscopic assistance. The lesion did not adhere to the surrounding nervous tissue, so en bloc removal was possible and appeared to be complete. A concave imprint of the lesion was still present. Summarily, the mass was left sided extramedullary and intradural without nerve root involvement. Pathological analysis showed a voluminous cystic lesion (approximated size of 10 mm × 6.5 mm) lined by a heterogeneous epithelium (Fig. 4). A pseudostratified columnar epithelium with cylindrical cells with basal nuclei and round-shaped apical poles, evoking an enteral or respiratory epithelium, was observed (Fig. 5a). Structures such as cilia or microvilli were sometimes present at the apical pole (Fig. 6). Moreover, a stratified squamous epithelium evoking a Malpighian epithelium, but without keratin production, was observed (Fig. 5b). Finally, a transitional epithelium similar to urinary epithelium was present. Mucus production was established by histochemical assays utilizing periodic acid-Schiff (Fig. 7a and b) and alcian blue (Fig. 7c). This highlighted the secretory nature of the epithelium. All of these different epithelia lacked differentiation. Immunohistochemical labelling was carried out. The wall of the cyst showed antigens of cytokeratin. This finding was consistent with the epithelial nature of the tissue. The cystic epithelial cells also stained positively for carcinoembryonic antigen (Fig. 8). This finding was consistent with an endodermal origin of the cyst. In contrast gliofibrillar acid protein and vimentine antigens were not expressed. Mucus production and the morphology of some cells with microvilli at the apical pole were also highly in favor of an endodermal origin of the cyst. The association of a cystic structure with heterogeneous epithelia of endodermal origin led to the diagnosis of a spinal neurenteric cyst, as described in humans. Post-operatively, neurological deficits dramatically worsened; the patient was paraplegic with loss of nociception in the left hind limb. Deep pain sensibility recovered after 5 days and the capacity to walk after 10 days. Fecal and urinary continence recovered after one month, and proprioceptive deficits were the only remaining neurologic deficit after six months. A follow-up MRI study performed 111 days after the surgery revealed no recurrence of the lesion and no spinal cord compression. No recurrence of clinical signs has been observed 18 months after surgery.

## Discussion

Intradural extra-axial “cystic or tumor-like lesions” of the thoraco-lumbar spinal cord are uncommon in young dogs. Histopathological features of epidermoid or dermoid cysts, nephroblastomas or Tarlov cysts are well described [3, 4]. In the present case, the cyst was lined by different types of epithelia that suggested a respiratory, digestive, cutaneous or urinary origin. Structures similar to cilia or a brush border at the apical pole of cuboidal cells and mucus secretion suggested a respiratory or digestive epithelium. The stratified squamous epithelium was similar to a cutaneous epithelium but with no sign of keratinization, in contrast to an epidermoid cyst. Moreover the endodermal origin was supported by the presence of mucus and microvilli at the apical pole of some cells and immunhistochemistry. In humans, a neurenteric cyst, also called an enterogenous cyst or endodermic cyst, is a congenital lesion characterized by a mucus-secreting epithelium mimicking the epithelium of the gastrointestinal tract [5]. Neurenteric cysts result from inappropriate partitioning of the embryonic notochordal plate and presumptive endoderm [6]. A failure during embryogenic development could be responsible for the persistence of an abnormal communication between the endoderm and neuroectoderm at 3 weeks of development in the human embryo [5]. Several theories have been proposed to explain the occurrence of neurenteric cysts [7]. For example Mac Donald et al. [8] proposed different mechanisms in which neurenteric cysts may develop: a primary adhesion of endoderm anterior to the notochord, incomplete excalation of the notochord, persistence of the neurenteric canal or formation of an accessory neurenteric canal with a split notochord, and displacement of endodermal cells. Bentley and Smith [9] postulated that the splitting of the notochord is the primary event in this pathology. The subsequent deficiency in the overlying neural plate could allow for an endodermal diverticulum to herniate through the spinal column and make contact with the surface ectoderm. In such a situation, the persistence of the neurenteric connection may be transient or permanent, partial or complete. In the canine fetus, closure of the neural tube occurs at about 20 days, so that we can expect that the neurenteric cyst formation occurs at this period of development in dogs [10].

A heterotopic epithelium reminiscent of gastrointestinal and respiratory tissue lead to a compressive cystic lesion in the pediatric and adult spine. Histopathological analysis of neurenteric tissue reveals a highly characteristic structure of columnar or cuboidal epithelium with or without cilia and mucus globules, as in the present case. Wilkins and Odum described 3 types of neurenteric cysts [11]: type A cysts contain either columnar or cuboidal cells, with ciliated and nonciliated components atop a basal membrane composed of type IV collagen. Type B cysts include all of the features of type A as well as additional tissue that may include bone, cartilage, lymphatic tissue, fat, or glandular components. Type C cysts are identified by type A features in association with ependymal or glial tissue. We think we can classify the neurenteric cyst in the present case as a type A. Spinal neurenteric cysts account for 0.7-1.3 % of spinal axis “tumors” in humans [12]. Neurenteric cysts are usually intradural and extramedullary lesions [6], as in the case reported here. According to the literature, the most common location is the cervicodorsal region, ventral to the spinal cord [13, 14]. In the thoracolumbar region, the neurenteric cyst appears to be situated dorsal to the spinal cord [14]. In our case, the cyst was in a dorso-lateral position in relation to the spinal cord. Human patients are typically symptomatic in the second and third decades of life, with a male predominance (sex ratio = 2) [5], but the reported age of presentation varies from 5 weeks to 52 years [13]. In this case, the dog was also a young adult. Myelopathic and/or radicular signs are size and location-dependent [12]. Pain is the main presenting clinical sign, not neurological deficits, which reflects the slow growth of the cyst [14]. In our case, massive spinal cord compression without dramatic neurological deficits supports the hypothesis of a slow-growing lesion. MRI appears to be the best modality for identifying the complex anatomy of spinal cysts [13, 15]. The cyst appears iso-intense on T1-weighted sequences and hyper-intense on T2-weighted sequences with no contrast enhancement. Edema surrounding the spine is generally not observed [15, 16]. These MRI features are considered typical of a neurenteric cyst, but variations can occur. In our case, the cyst presented the characteristic MRI pattern of a neurenteric cyst. Radiology and computed tomography can also be useful tools for the evaluation of osseous malformations associated with this lesion [12]. Indeed in a case series of 23 human patients with neurenteric cyst, 87 % showed vertebral anomalies at the same level as the cyst on radiographic evaluation [17].

In humans, immunohistochemistry is key to confirming the endodermic origin of the lesion. Cystic epithelial cells typically stain negatively for gliofibrillar acid protein (GFAP), neuronal specific enolase, vimentine, S100 antibodies, and positively for cytokeratin epithelial membrane antigen and carcinoembryonic antigen (CEA) [6, 12]. Positive CEA staining supports a theory of shared lineage between the cystic wall and the intestinal mucosa. In the case of intramedullary lesions, astrocytes within the cyst wall may stain positively for GFAP in comparison to the typical negative staining pattern of extramedullary cysts [18].

Total surgical excision led to a good, although progressive, neurological recovery in our patient. Surgical removal is the treatment of choice in humans [16], resulting in minimal morbidity [13]. Total removal seems to be curative, but recurrence rates as high as 37 % have been reported after incomplete resection due to factors such as cyst adhesion to surrounding structures and unclear dissection planes [12].

## Conclusion

We report an unusual intradural extramedullary cyst in a 2-year-old female crossbreed dog. The MRI study and histological findings are consistent with an intradural neurenteric cyst, as described in humans. The neurenteric cyst should be included in the differential diagnoses for tumor-like or cystic intradural lesions in the young dog. Prognosis for this type of cyst seems to be good, as total surgical removal led to progressive clinical improvement with no recurrence at 18 months.
